# UFM1 at the endoplasmic reticulum: linking ER stress, ribosome quality control, and ER-phagy

**DOI:** 10.1042/EBC20253054

**Published:** 2025-10-09

**Authors:** Masaaki Komatsu, Gaoxin Mao

**Affiliations:** 1 Department of Physiology, Juntendo University Graduate School of Medicine, Japan; 2 Autophagy Research Center, Juntendo University Graduate School of Medicine, Japan

**Keywords:** encephalopathy, endoplasmic reticulum, proteostasis

## Abstract

Ubiquitin-fold modifier 1 (UFM1) is a small protein that functions as a ubiquitin-like modifier attached to other proteins to alter their behavior. Although less famous than ubiquitin, UFM1 has gained attention as a key regulator of proteostasis (protein homeostasis) in the cell. Notably, the endoplasmic reticulum (ER) has emerged as the central stage for UFM1’s activity. UFM1 was initially recognized for its role in the ER stress response, and we now know it orchestrates two critical quality-control processes at the ER: ribosome-associated quality control and selective autophagy of the ER. Together, these mechanisms ensure that the cell can cope with misfolded proteins and stalled ribosomes, maintaining the health of the ER and the proteins it produces. In this review, we will explore how UFM1 works at the ER, how its components are regulated during stress, how it facilitates both immediate quality control and longer-term ER turnover, and how disruptions in this system lead to disease, especially in the nervous system.

## Introduction

Post-translational modifications are critical for dynamically regulating protein function, localization, and interactions. Among these, ubiquitin and ubiquitin-like modifiers such as SUMO, NEDD8, and ISG15 play central roles in modulating proteostasis and cellular stress responses [1,2]. Discovered in 2004, ubiquitin-fold modifier 1 (UFM1) has since emerged as a pivotal ubiquitin-like regulator that safeguards endoplasmic reticulum (ER) homeostasis. Unlike the canonical ubiquitin system, UFM1 is conjugated to a relatively small set of substrates and operates primarily at the ER, where it modulates protein synthesis, folding, and degradation pathways [3–5].

The ER is a crucial site for the synthesis of membrane-bound and secretory proteins, and its integrity is maintained by elaborate surveillance systems. When translation stalls or misfolded proteins accumulate, the ER activates mechanisms such as the unfolded protein response (UPR), ribosome-associated quality control (RQC), and selective autophagy of ER fragments (ER-phagy) [6–9]. UFM1 plays a pivotal role in these processes by tagging specific substrates for downstream processing, acting through its dedicated E1, E2, and E3 enzymatic cascade.

This review summarizes our current understanding of UFM1 function at the ER. I begin by describing the subcellular localization and recruitment of UFM1-conjugating enzymes, then examine how UFM1 pathway components are transcriptionally induced under ER stress. I further explore how UFM1 co-ordinates ER-associated ribosome quality control (ER-RQC) and ER-phagy, and conclude by discussing the pathological consequences of UFM1 dysregulation, particularly in the nervous system.

## Site of UFM1 action: the ER as the central hub of UFMylation

**Figure 1:**
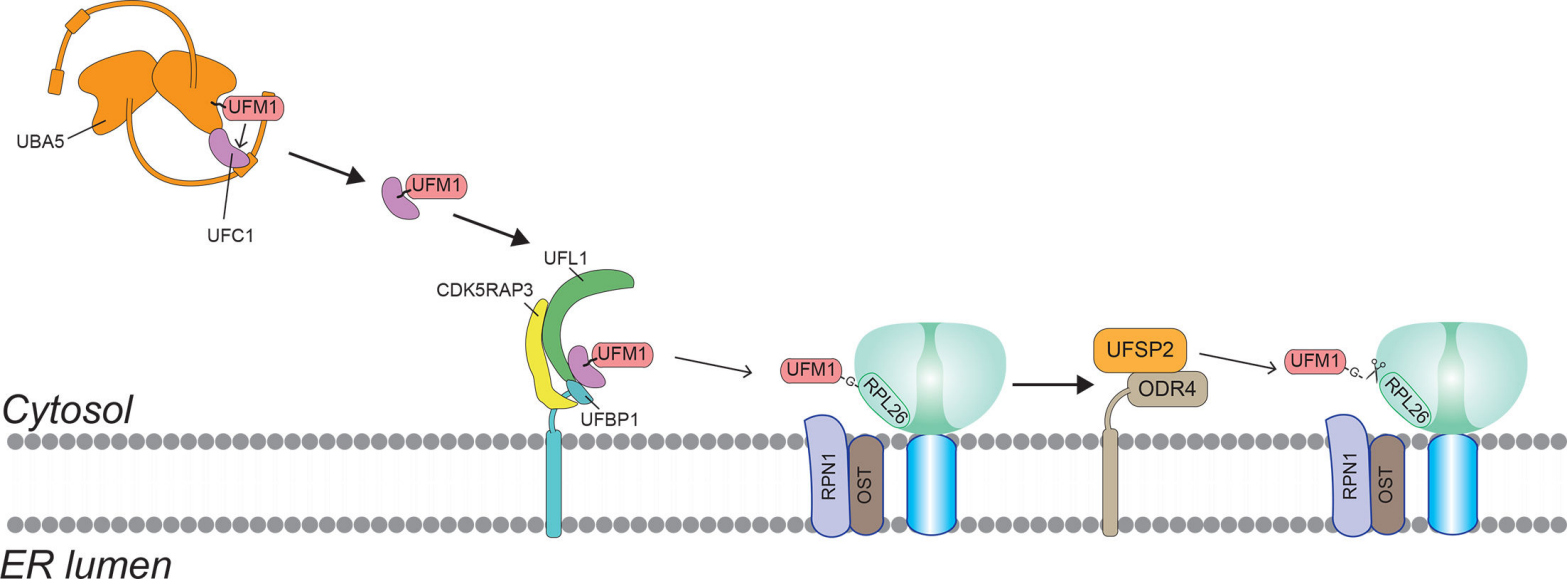
The UFM1 system on the ER. Schematic overview of the UFM1 conjugation and deconjugation toolkit at the endoplasmic reticulum. UBA5 activates UFM1 and transfers it to UFC1; the UFL1–UFBP1 scaffold E3 complex anchors to the ER membrane, where CDK5RAP3/C53 modulates substrate selection. Key substrate, a ribosomal protein RPL26 is indicated, together with the deUFMylase UFSP2 and its ERtether ODR4.

The ER is the primary site where UFM1 executes its functions. This is largely due to the localization of UFM1’s conjugation (attachment) and deconjugation machinery at the ER (Figure 1). UFM1 is attached to target proteins through a dedicated enzyme cascade, analogous to ubiquitination but with its own specialized enzymes. The cascade begins with UBA5, the E1 activating enzyme, which uses ATP to activate UFM1 [10]. Activated UFM1 is then transferred to UFC1, the E2 conjugating enzyme [10]. Finally, UFM1 is covalently ligated to a substrate protein by the E3 ligase complex, chiefly composed of UFL1 (also known as RCAD) together with its cofactors [11,12]. This E3 ligase complex is unusual in that it operates as a scaffold and lacks the typical RING/HECT domains found in many ubiquitin E3 enzymes. Instead, UFL1 relies on two partner proteins, UFBP1 and CDK5RAP3, to carry out UFM1 transfer with high specificity [12].

UFBP1 (DDRGK1) is integral to the E3 complex’s localization and function. It contains a transmembrane helix that anchors the UFM1 ligase complex directly to the ER membrane [11]. By tethering UFL1 to the ER, UFBP1 effectively positions the UFM1-conjugating machinery right where it’s most needed: at the cytosolic face of the ER [13,14], where ribosomes dock to synthesize secretory and membrane proteins. UFBP1 also contains a UFM1-interacting motif that helps bind UFM1 or UFMylated proteins [15], suggesting it not only localizes the ligase but also aids in recognizing UFM1 modifications. The second cofactor, CDK5RAP3 (also known as C53 or LZAP), binds to the UFL1–UFBP1 complex and plays a regulatory role. While not required for the basic UFM1 ligation activity in a test tube, CDK5RAP3 is important inside cells for fine-tuning which substrates get UFMylated [12,16]. It can modulate substrate selection by, for example, preventing the ligase from tagging UFBP1 itself at a particular site (UFBP1 Lysine-267) until the appropriate time [15] and by directing the UFMylation of RPL26 [11,12]. In this way, CDK5RAP3 acts as a kind of switch or guard on the ligase complex. Moreover, as we will see later, CDK5RAP3 has a second life as an autophagy receptor when ER stress is high [17–19], underscoring its dual function in the UFM1 system.

In addition to these ER-anchored ligase components, recent studies have shown that UBA5 is also targeted to the ER via interaction with the autophagy-related protein GABARAP [20,21]. UBA5 contains an atypical LC3-interacting region (LIR) motif at its C-terminus that binds specifically to GABARAP and GABARAPL2, but not to LC3 proteins [21]. This binding allows cytosolic UBA5 to associate with ER membranes independently of classical autophagy [20,21]. This recruitment is essential for positioning UBA5 near its downstream partners and ensuring efficient UFMylation of ER-resident substrates. Disruption of this interaction – either by deletion of GABARAP subfamily members or mutation of UBA5’s LIR motif– reduces UFMylation levels at the ER and impairs ER-associated quality control.

Because UFBP1 anchors the E3 ligase at the ER, the targets of UFM1 modification (UFMylation) are primarily ER-associated proteins. Indeed, one of the first identified substrates of UFM1 is a ribosomal protein (RPL26) on the large 60S ribosomal subunit that attaches to the ER during protein translation [22,23]. Other substrates include ER-resident proteins like ribophorin 1 (RPN1) and certain ER enzymes [19,24,25] – all of which localize to the ER network. This localization bias means UFM1’s effects are largely concentrated on processes happening at the ER (such as protein synthesis and folding). Additionally, the removal of UFM1 from proteins – carried out by specialized proteases called deUFMylases – is also focused at the ER. Two proteases, UFSP1 and UFSP2, can cleave UFM1 off of substrates [26–28]. UFSP2, the major deUFMylating enzyme in most human cells, lacks a membrane anchor itself but is recruited to the ER membrane by associating with an ER protein called ODR4 and/or UFBP1 [29,30]. This ensures that UFM1 removal (deconjugation) occurs in the same vicinity. By co-localizing the ‘writers’ (UFM1 ligases) and ‘erasers’ (UFM1 proteases) at the ER, the cell tightly controls UFM1 signals exactly where needed and prevents unwanted UFMylation elsewhere. How the E3 ligase complex and UFM1-specific proteases co-ordinate their activities to form a complete UFM1 cycle remains an open question for future investigation. In summary, the ER acts as the central hub for UFM1 activity, with UFM1’s entire toolkit – from the enzymes that attach it to substrates to those that remove it – strategically stationed at the ER membrane. This strategic placement enables UFM1 to specifically monitor and respond to the challenges of ER function.

## Transcriptional regulation of the UFM1 pathway components during ER stress

Cells not only position the UFM1 machinery at the ER, but they also adjust the levels of UFM1 and its enzymes in response to stress. When the ER is under duress – for instance, when misfolded proteins accumulate in the ER lumen – cells activate a protective program known as the UPR). The UPR triggers a broad change in gene expression to help the cell cope with ER stress, including enhancing protein folding capacity and reducing overall protein synthesis [31,32]. As part of this adaptive response, the genes encoding the UFM1 system are themselves up-regulated.

Multiple studies have shown that ER stress leads to increased transcription of UFM1 and its pathway components. For example, pancreatic β cells (which have a high secretory burden and a sensitive ER) ramp up production of UFM1, UFBP1, and UFL1 when subjected to ER stress [33]. In other mammalian cells as well, treating with ER stress-inducing agents causes the mRNA levels of UFM1 and UFL1 to rise. This co-ordinated induction suggests that UFMylation is an integral branch of the cell’s stress relief strategy. In fact, one of the key UPR transcription factors, XBP1, directly controls UFM1 pathway genes. The promoter region of the UFM1 gene contains a site for XBP1, and experiments have confirmed that active XBP1 (produced during UPR) binds there to drive UFM1 gene expression [34]. Likewise, XBP1 helps induce other components like UBA5 (E1), UFL1 (E3), and CDK5RAP3 under stress conditions [34]. If XBP1 is genetically knocked out, cells fail to up-regulate UFM1 and its enzymes in response to ER disturbances – highlighting that this transcriptional circuit is UPR-dependent.

Why would the cell do this? The induction of the UFM1 system under stress makes perfect sense considering UFM1’s role in maintaining ER function [3,4]. By boosting the levels of UFM1 and its conjugation machinery, the cell prepares itself to handle an increased load of misfolded proteins or stalled ribosomes that come with ER stress. In essence, UFM1 becomes a reinforcer of ER quality control when the going gets tough. Therefore, if the UFM1 system is impaired, cells often exhibit signs of ER stress even under normal conditions. Knocking down UFM1 or its ligases in cultured cells can spontaneously activate the UPR and even cause the ER to swell (as the cell tries to compensate by expanding the ER volume) [24,35–38]. This indicates that a baseline level of UFMylation is required to keep the ER running smoothly. Thus, through UPR-driven transcriptional regulation, cells ensure UFM1 pathway components are on hand to bolster ER defenses when needed most, integrating UFMylation into the broader network of ER stress responses.

## UFM1 in ER-associated translational quality control and ER turnover

**Figure 2:**
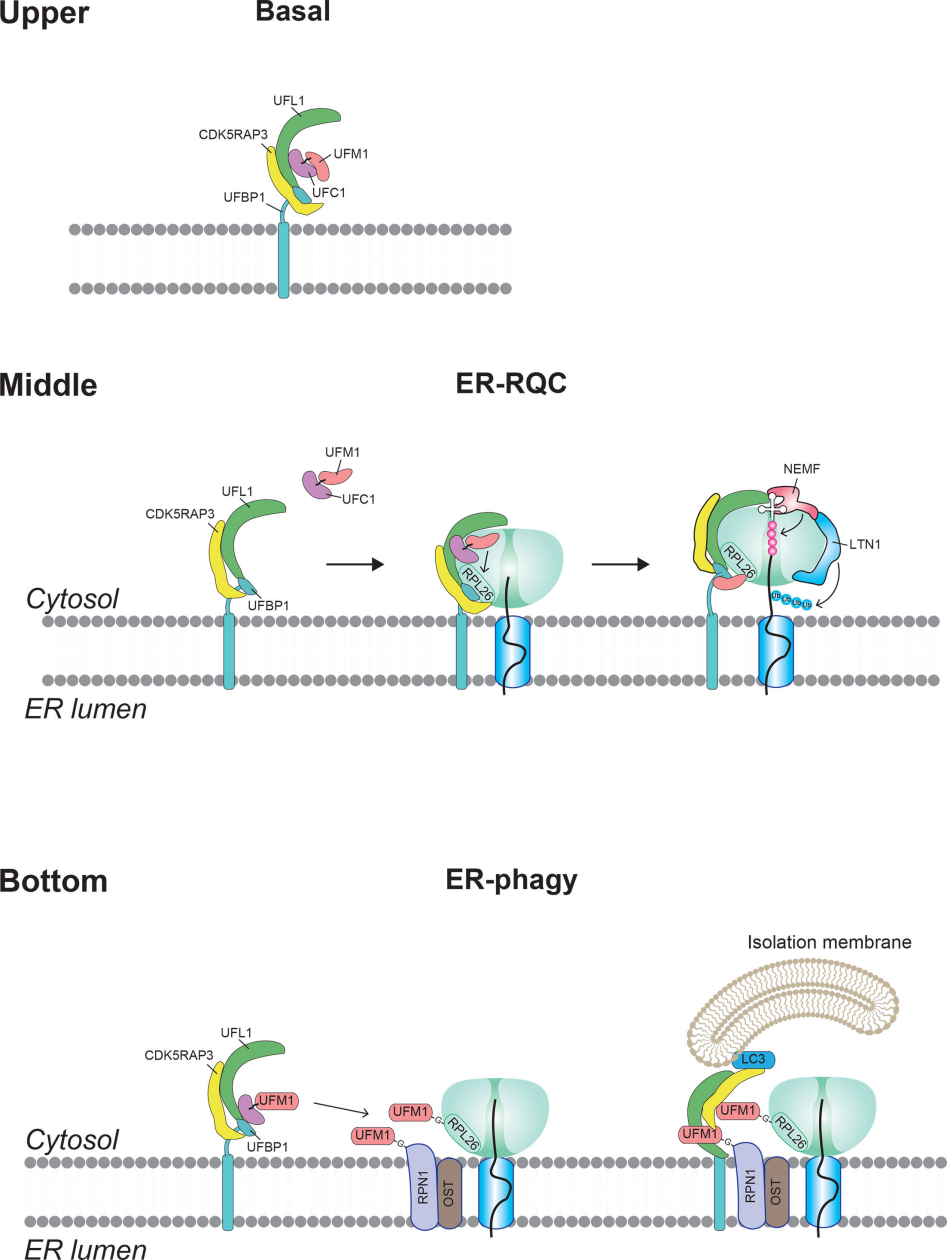
UFM1-mediated quality control functions of the ER. Illustration of how UFM1 safeguards ER proteostasis. Upper: under basal conditions, CDK5RAP3 binds the UFL1–UFBP1 complex and holds UFM1 in an autoinhibited state. Middle: upon ribosome stalling, RPL26 is UFMylated, prompting release of the 60 S subunit from the Sec61 translocon and recruitment of cytosolic RQC factors (LTN1/NEMF). Bottom: when ER stress persists, CDK5RAP3 switches to an ERphagy receptor by engaging LC3/ATG8, facilitating selective autophagic sequestration of damaged ER domains. Major signalling branches of the unfolded protein response (PERK, IRE1α, and ATF6) and their transcriptional up-regulation of UFM1 pathway genes are annotated for context.

Growing lines of evidence indicate that the UFM1 system plays pivotal roles in both ER-RQC and ER-phagy (Figure 2). These processes act in concert to resolve stalled translation events at the ER and to remove persistently damaged ER regions, thereby maintaining ER proteostasis.

### Ribosome stalling and UFM1-mediated ER-RQC

During translation at the ER, ribosomes can stall if an mRNA is faulty or a nascent chain becomes stuck in the translocon. In ER-RQC, the RQC factor NEMF marks the stalled nascent chain by CATylation, adding C-terminal alanine/threonine residues. The UFM1 E3 ligase complex (UFL1–UFBP1–CDK5RAP3) then modifies the ribosomal protein RPL26 with UFM1, licensing the 60S subunit to detach from the Sec61 channel. This exposes the aberrant nascent chain to LTN1, an E3 ubiquitin ligase that tags it for proteasomal degradation. Through this sequence, UFM1 ensures stalled ribosomes are rapidly recycled and potentially toxic proteins are eliminated. Recent cryo-EM structural analyses have uncovered the basis for how the UFM1 E3 ligase complex and RQC factors co-operate on ER-bound 60S ribosomal subunits [13,14,39], revealing a co-ordinated mechanism that links RPL26 UFMylation to ribosome–translocon dissociation and subsequent processing of stalled nascent chains. The structural basis of this mechanism is described in detail in Komatsu M, Noda NN, Inada T., Nat Rev Mol Cell Biol, in press [40]; the following provides an overview.

How does UFM1 help? The attachment of UFM1 to the ribosome prompts a series of actions that free the stalled machinery (Figure 2 middle). Structural studies show that once UFM1 is conjugated to RPL26, the UFM1 ligase complex itself stays bound to the ribosome, almost clamping onto it. This ‘clamp’ blocks certain sites on the ribosome (like where tRNAs bind and where the protein exits) and helps pry the ribosome away from the ER translocon (Sec61) [13,14]. Recent cryo-EM analyses have further revealed that, on stalled ER-bound 60S particles, the C-terminal domain of UFL1 adopts a rotated conformation that avoids steric clash with the RQC factor NEMF, while a loop domain of UFL1 forms a β-augmented interface with the NFACT-C domain of NEMF [39]. This interaction stabilizes NEMF in a conformation that pauses CATylation, potentially preventing premature peptidyl-tRNA cleavage. Based on sequential structural snapshots, a temporal order for ER-RQC has been proposed: (1) NEMF engages SEC61-bound 60S (CATylation-competent), (2) E3UFM1 binds and catalyzes UFMylation of RPL26/uL24, (3) UFMylation stabilizes DDRGK1 at the tunnel exit and promotes dissociation of the 60S from the translocon, enabling LTN1 recruitment and ubiquitylation of the arrested polypeptide, and (4) following deUFMylation by UFSP2, E3UFM1 dissociates, allowing peptidyl-tRNA cleavage and extraction of the ubiquitylated ER-arrested peptide for proteasomal degradation. In other words, UFMylation of RPL26 triggers the release of the ribosome from the membrane channel, which is a necessary step to then deal with the jammed protein. With the ribosome detached from the ER membrane and stalled translation terminated, traditional quality control factors in the cytosol can now assess the situation – an enzyme called LTN1 (an E3 ubiquitin ligase) tags the aberrant nascent polypeptide with ubiquitin so that it gets degraded by the proteasome [41,42]. In the absence of UFM1, this whole process falters: stalled ribosome complexes remain stuck at the ER membrane with Sec61, and the unfinished proteins accumulate, stressing the cell [22,23,43]. Thus, UFM1 acts as a linchpin that connects the detection of a ribosomal stall to the actual resolution – it licenses the ribosome for recycling and the faulty protein for destruction. The UFM1 E3 complex essentially switches from being a ‘writer’ that adds UFM1 to the ribosome into a ‘reader’ that holds onto the UFMylated ribosome and orchestrates the hand-off to downstream quality control.

It’s worth noting that UFM1’s role in translational quality control is specific to the context of the ER. We call this ER-RQC to distinguish it from general cytosolic RQC. The specificity comes from UFBP1 anchoring the ligase to ER-bound ribosomes and from the selectivity of UFM1 for RPL26 only when ribosomes are docked on the ER [13–15]. This ensures that regular cytosolic translation uses the standard ubiquitin-RQC pathway, whereas ribosomes translating secretory proteins get an added layer of protection via UFM1. This specialization is critically important in cells with a high secretory load, like neuronal cells or secretory glands, where even a single stalled ribosome at the ER could cause a traffic jam of protein production. By promptly ejecting stalled ribosomes and clearing faulty products, UFM1-mediated RQC preserves the flow of translation and protein folding at the ER.

### Overload-induced ER-phagy: using autophagy to clear the ER

While ribosome-quality control deals with individual stalled ribosomes and nascent proteins, sometimes the ER can be overwhelmed with misfolded proteins or extensive damage. In such cases, cells may resort to a more drastic, but necessary, measure: removing portions of the ER itself. This selective degradation of ER fragments via the autophagy-lysosome pathway is called ER-phagy (or reticulophagy) [7,44]. Intriguingly, the UFM1 system that handles immediate quality control also plays a major role in initiating ER-phagy when the ER is overloaded [15,17,18,24] (Figure 2 bottom). Think of it as a two-tier response: UFM1 first tries to fix problems on the spot (via ER-RQC), but if the problems persist or multiply, UFM1 helps trigger the cleanup of the area by autophagy.

Selective autophagy of organelles, like mitophagy for mitochondria, usually requires a tagging system and a receptor. In mitophagy, damaged mitochondria get tagged by ubiquitin (through PINK1/Parkin), and autophagy receptors like NDP52 bind those tags and the autophagy machinery (LC3 proteins) to engulf the mitochondria [45]. By analogy, in UFM1-mediated ER-phagy, UFM1 serves as the tag that marks parts of the ER for destruction. Under conditions of severe or prolonged ER stress – such as many ribosome stalls or an accumulation of misfolded proteins that the proteasome can’t handle – the UFM1 E3 ligase modifies certain ER-associated substrates as a signal for ‘this area needs removal.’ Key substrates identified include RPN1, a component of the oligosaccharyltransferase complex embedded in the ER, and RPL26 on ribosomes [24]. UFMylation of these targets is like putting a sticker on that ER region indicating it’s damaged or overloaded.

The next crucial piece is the autophagy receptor that recognizes this UFM1 tag and connects the tagged ER to the autophagosome formation process. Here, CDK5RAP3 (C53) re-enters the story in its second role. Under normal growth conditions, CDK5RAP3 binds to the UFM1 ligase complex (UFL1–UFBP1–CDK5RAP3) [12,15] and, via its long loop region containing shuffled ATG8-interacting motifs (sAIMs), can also bind UFM1 [18]. During ER stress, especially when multiple ribosome stalls occur, ER-associated substrates such as RPL26 and RPN1 become UFMylated. CDK5RAP3 then recognizes these UFM1-modified substrates and, through a separate sAIM, engages ATG8/LC3 [17,18]. This dual-binding ability enables efficient recruitment of UFMylated ER regions to form autophagosomes, thereby promoting ER-phagy. Loss of CDK5RAP3 has been shown to markedly reduce ER stress-induced ER-phagy [17,18].

CDK5RAP3 bridging the gap means that the patch of ER containing UFM1-tagged cargo is brought in contact with the forming autophagosome. CDK5RAP3 simultaneously can bind UFM1 (on the tagged ER) and LC3 (on the autophagosome membrane) [17,18,24], acting as a tether. This allows a segment of the ER – including any problematic ribosome-nascent chain complexes and misfolded proteins in that vicinity – to be sequestered into a double-membrane vesicle (an autophagosome). The autophagosome will then close and eventually fuse with a lysosome, leading to degradation of its contents, effectively pruning the damaged section of ER. Notably, experiments have found that if CDK5RAP3 is removed or mutated, ER-phagy in response to stress is impaired, even though general autophagy (like that induced by nutrient starvation) might still occur via other receptors [17,18]. This indicates that UFM1-CDK5RAP3 represents a stress-specific branch of ER-phagy.

The UFM1-driven ER-phagy mechanism has been likened to a safety valve. It is triggered particularly by overload conditions – for instance, when there are so many stalled ribosomes that simply degrading the individual proteins isn’t enough, or when misfolded aggregates start forming in the ER membrane. Under these conditions, clearing out an entire ER subdomain is more efficient. UFM1’s role is to mark that subdomain, and CDK5RAP3’s role is to haul it away to the lysosome. Additional UFM1 targets keep emerging; for example, an ER enzyme called CYB5R3 (cytochrome b5 reductase) can be UFMylated and sent to lysosomes [19], indicating that UFM1 can tag not just ribosomes but also other ER resident proteins for autophagic turnover. Through such targeted tagging, UFM1 doesn’t just indiscriminately cause wholesale ER digestion; instead, it fine-tunes which parts of the ER (or which specific proteins) to remove, thereby maintaining quality control without sacrificing too much of the ER’s functional capacity. However, compared with ER-RQC, analyses of ER-phagy – particularly those addressing its structural basis – remain limited, and many aspects of how the UFM1 system regulates ER-phagy are still poorly understood. Future studies should aim to elucidate the detailed structural and dynamic mechanisms underlying substrate recognition and the switch to ATG8/LC3 engagement.

In summary, UFM1 has a dual contribution to ER maintenance: first, by ensuring stalled ribosomes and aberrant proteins are handled immediately via the proteasome (ER-RQC), and second, by marking persistently problematic areas of the ER for removal via autophagy (ER-phagy). These processes are co-ordinated – the same UFM1 system and players (UFL1, UFBP1, and CDK5RAP3) are involved in both – which allows the cell to seamlessly escalate its response from a localized fix to a broader cleanup as needed.

## Pathological implications: UFMylation, neurodevelopment, and neurodegeneration

Given UFM1’s critical role in maintaining ER protein folding and translation quality, it’s no surprise that disruptions in this pathway can lead to disease. In fact, the importance of the UFM1 system first became evident when researchers knocked out UFM1 or its key enzymes in mice – the mice exhibited severe phenotypes like embryonic lethality or organ failure [3], underlining that cells cannot develop properly without UFMylation. In humans, mutations in components of the UFM1 pathway have been linked to early-onset neurological disorders and other pathologies [4,5].

One striking set of findings comes from rare genetic diseases in children. Several families have been identified where babies are born with biallelic (both copies) loss-of-function mutations in UFM1 or its enzyme genes (such as UBA5 or UFC1) [46–48]. These infants present with severe epileptic encephalopathy – a disorder of brain development characterized by refractory seizures, developmental delay, and often microcephaly (an abnormally small head/brain). Essentially, their neurons cannot develop and function normally. At the cellular level, cells from these patients show an inability to properly UFMylate substrates, which likely means their neurons are suffering chronic ER stress and failing to clear protein jams. Similarly, mutations in the gene encoding UFL1 (the UFM1 E3 ligase) or UFBP1 have been found to cause neurological symptoms or perinatal lethality in animal models [49], again pointing to an essential role in the brain. The nervous system may be especially vulnerable to defects in UFM1-mediated quality control because neurons are long-lived, highly active cells that rely on impeccable proteostasis. If ribosome quality control at the ER is compromised, neurons might accumulate toxic protein fragments or experience unresolved ER stress that triggers cell death pathways. Indeed, in mice that specifically delete UFM1, UFL1, or UFBP1 in the brain, there is massive neuronal loss and neurodevelopmental failure resembling the human mutations [47,49].

Beyond development, problems with UFM1 can also contribute to neurodegeneration later in life. Recent studies have connected the UFM1 system to the handling of proteins implicated in adult-onset neurodegenerative diseases. For example, tauopathies (like certain forms of dementia) and synucleinopathies (like Parkinson’s disease) are characterized by misfolded, aggregating proteins (τ and α-synuclein, respectively) that can overwhelm cellular quality control mechanisms. The UFM1 pathway has been shown to influence the propagation of these protein aggregates [50,51]. While the exact details are still being worked out, researchers found that if the UFM1 system is impaired, cells are less able to cope with toxic forms of τ or α-synuclein, leading to more accumulation and spread of these aggregates. Conversely, a well-functioning UFM1 system might help degrade or sequester these disease-related proteins, possibly through ER-phagy or related pathways, thus slowing disease progression. In this way, UFMylation appears to act as a suppressor of neurodegenerative pathology: it keeps proteostasis in check, and when it falters, neurons may succumb to proteotoxic stress.

Aside from the nervous system, UFM1 pathway defects can affect other organs as well. For instance, a loss-of-function mutation in UFBP1 [52] or certain mutations in UFSP2 (the deUFMylase) [53,54] cause a distinct hereditary disorder called Schochat-type spondyloepimetaphyseal dysplasia, which affects bone growth and development. This illustrates that UFM1’s role in ER homeostasis is also vital in highly secretory cells like chondrocytes (which produce collagen for bone and cartilage). Additionally, emerging evidence links UFMylation to metabolic diseases and cancer. In some cancers, the UFM1 machinery is dysregulated – sometimes turned down, other times up – in ways that help cancer cells cope with stress or, if disrupted, can hinder tumor growth. For example, loss of UFM1’s deconjugating enzyme UFSP2 can accelerate cell proliferation in colon cancer [55], while overexpression of UBA5 (the E1) in certain tumors might allow cancer cells to survive ER stress in the tumor environment [56]. Moreover, in the liver, deleting UFL1 or UFBP1 leads to uncontrolled cell growth and liver tumor formation [57], likely because a lack of UFMylation causes unmitigated ER stress and alters signaling pathways (like mTOR) that then drive cancerous changes.

The clearest thread through all these conditions – from brain development disorders to adult neurodegeneration to other diseases – is that when UFM1-mediated ER quality control is disrupted, cells cannot maintain ER homeostasis, and this can trigger cell dysfunction or death. In neurons, unresolved ER stress and accumulation of aberrant proteins contribute to neurodegeneration; in secretory cells like those in cartilage or pancreas, it leads to cell failure and tissue malformation or degeneration. The pathological outcomes underscore how fundamentally important UFM1 is: it’s not merely a stress-related footnote, but rather a central pillar of cell health.

This recognition has spurred interest in UFM1 as a potential therapeutic target or biomarker. If enhancing UFMylation can improve a cell’s ability to handle misfolded proteins, one might envision therapies to boost the UFM1 pathway in neurodegenerative diseases or aging-related proteostasis decline. Conversely, in contexts where UFM1 might be helping cancer cells survive therapy-induced stress, carefully dampening its activity could sensitize those cells to treatment. While no drugs currently modulate UFM1 specifically, ongoing research into its mechanism is setting the stage for future interventions.

## Conclusion

UFM1 has emerged as a guardian of the ER, co-ordinating rapid responses to ribosomal stalls and long-term responses to chronic stress by culling damaged ER. Its machinery is strategically centered at the ER, its production is dialed up when the ER is in trouble, and its failure is felt most acutely in cells that depend on a robust ER quality control system. The study of UFM1 has illuminated a sophisticated quality control axis in molecular cell biology – one that links the worlds of protein folding, translational control, autophagy, and human disease. As we continue to unravel UFM1’s roles, we gain not only fundamental insight into cellular homeostasis but also new avenues to understand and eventually treat diseases tied to ER dysfunction and protein mismanagement.

**Summary points**

- Ubiquitin-fold modifier 1 (UFM1) is a ubiquitin-like modifier that safeguards endoplasmic reticulum (ER) function by co-ordinating ER-associated ribosome quality control and autophagy of ER.
- The UFM1 conjugation system is spatially and transcriptionally regulated in response to ER stress.
- Defects in UFM1 signaling cause neurodevelopmental and degenerative diseases, highlighting its essential role in proteostasis.

## Open Access

This article has been published open access under our Subscribe to Open programme, made possible through the support of our subscribing institutions, learn more here: https://portlandpress.com/pages/open_access_options_and_prices#conditional

## Abbreviations

CDK5RAP3: CDK5 regulatory subunit-associated protein 3
DDRGK1: DDRGK domain-containing protein 1
ER: endoplasmic reticulum
ER-RQC: ER-associated ribosome quality control
ER-phagy: autophagy of ER
GABARAP: Gamma-Aminobutyric Acid Receptor-Associated Protein
GABARAPL2: Gamma-Aminobutyric Acid Receptor-Associated Protein-Like 2
ISG15: Ubiquitin-like protein ISG15
LC3: Microtubule-Associated Protein 1 Light Chain 3
LIR: LC3-interacting region
LTN1: E3 ubiquitin-protein ligase listerin
LZAP: Leucine Zipper-Containing ARF-Binding Protein
NEDD8: Neural precursor cell Expressed, Developmentally Downregulated protein 8
NFACT: NEMF-Associated Factor
ODR4: Protein odr-4 homolog
ODR4: Odorant Response Abnormal Protein 4 Homolog
RCAD: Regulator of C53 and DDRGK1
RING/HECT: Really Interesting New Gene / Homologous to E6AP C-Terminus
RPL26: Large ribosomal subunit protein uL24
RPL26: 60S Ribosomal Protein L26
RPN1: ribophorin 1
RQC: ribosome-associated quality control
SEC61: Protein Transport Protein SEC61 (α/β/γ) Subunit
SUMO: Small ubiquitin-related modifier 1
UBA5: Ubiquitin-like modifier-Activating Enzyme 5
UFBP1: UFM1-binding and PCI domain-containing protein 1
UFL1: E3 UFM1-Protein Ligase 1
UFM1: ubiquitin-fold modifier 1
UFSP2: UFM1-specific protease 2
UFSP: UFM1-Specific Protease
UPR: unfolded protein response
XBP1: X-Box Binding Protein 1
sAIMs: shuffled ATG8-interacting motifs
uL24: 60S Ribosomal Protein L24
